# The Origin of Hospitals

**Published:** 1887-06-04

**Authors:** 


### The Origin of Hospitals.

The following contributions to the history of the institution of hospitals we select from replies sent in to competition No. 18, Vol. I:—

"Hospital" is derived from the Latin *hospes*, a guest, and *hospitium*, hospitality. It afterwards meant a guest-chamber and an inn, and in mediæval Latin, a palace. "*Actium apud hospitale juxta Corboleum anno Domini* MCCXLIII." "Hospital," with its present signification, is an outcome of Christianity, and was altogether unknown to the ancients. The first hospitals, or *hospices*, were resting-places for pilgrims, the best known being that of St. John's, at Jerusalem. The religious orders, following the precepts of their Divine Master, added guest-chambers to their houses for the sick and poor. As the temporal power of the Church declined, these institutions were taken up by the State, or endowed by private benevolence: in our own country by the latter. Hospitality is freely offered, unchecked by State interference, and limited only by amount of funds. *Floreat caritas.*—Condorcet.

It is interesting to trace the modification of meaning from the old Latin word *hostis*, an enemy, to our English word hospital, now bearing so friendly a signification. *Hostis* contains the root of the words *hospes* and *hospitium*, etc., etc., in Latin; host and hospital, etc., etc., in English. So that we find in it both an important modification and an amplification, as *hostis* (enemy) may be synonymous with foreigner or stranger, while *hospes* and *hospitium* contain the idea of the entertainment of strangers. While, again, we have the early use of the English word "hospital" applied similarly to an inn—

> "A goodly castle plac'd
> Forby a river, in a pleasant dale;
> Which choosing for that evening's hospital,
> They thither march'd."—Spenser.

And then later a richer and fuller meaning attaches to it as a place where hospitality is offered freely and fully to the sick and suffering, through the interest and generous sympathy of their more favoured brethren; where our skilful doctors are the hosts, and our trained nurses are the handmaidens to the worn and wearied guests; where those who enter in do not have to "abandon hope," but finding there help and healing for body and mind, find too a fresh measure of hopefulness for the future.—Mater.

"Hospital," from the Latin *hospitalis*=hospitable. The term was applied originally to a place of entertainment for travellers and pilgrims—

> "A goodly castle plac'd
> Forby a river, in a pleasant dale;
> Which choosing for that evening's hospital,
> They thither march'd."—Spenser.

Later on the name was given to any building set apart for the reception of persons unable to supply their own wants, and who were more or less dependent on public help. At the present time it is applied (with a few exceptions, as Christ's Hospital, school for boys, etc.) to those institutions where sick people, chiefly the poor, are taken in and cared for during their time of illness, and where they have the benefit of the best surgical and medical skill, and nursing.—Gip.

Hospital: Norman French; hospital, from Latin adjective, *hospitalis.* The old sense is any place for shelter or entertainment. So in Spenser's *Faerie Queene*—

> "They spy'd a goodly castle plac'd
> Forby a river in a pleasant dale;
> Which choosing for that evening's hospital,
> They thither march'd."

This having become obsolete, the word only survives now as a place built for the reception of the vile, a support of the poor. Latham Johnson gives from Sir H. Wotton:—"They who were so careful to bestow them in a college when they were young would be so good as to provide for them in some hospital when they were old." And from Addison:—"I am about to build an hospital, which I will endow handsomely for twelve old husbandmen." Presumably these passages show the time at which the word became restricted to its modern sense. It is to be noticed that Christ's Hospital is only one familiar instance of a large number which may serve to remind one that the restriction of the word to denote a place of sick tendance only is quite unnecessary. There seems to be nothing more calling for notice, only it may be observed that in Old English the adjective "hospital"=hospitable, is to be found.—Ostrich.

